# Completely Plant-Based Diets That Meet Energy Requirements for Resistance Training Can Supply Enough Protein and Leucine to Maximize Hypertrophy and Strength in Male Bodybuilders: A Modeling Study

**DOI:** 10.3390/nu16081122

**Published:** 2024-04-10

**Authors:** David M. Goldman, Cassandra B. Warbeck, Micaela C. Karlsen

**Affiliations:** 1 Department of Public Health, University of Helsinki, 00014 Helsinki, Finland; 2 Department of Research and Development, Metabite Inc., New York, NY 10036, USA; 3 Department of Family Medicine, University of Alberta, Edmonton, AB T6G 2R3, Canada; cwarbeck@ualberta.ca; 4 Department of Research, American College of Lifestyle Medicine, Chesterfield, MO 63006, USA; mkarlsen@lifestylemedicine.org; 5 Departments of Applied Nutrition and Global Public Health, Adjunct Faculty, University of New England, Biddeford, ME 04005, USA

**Keywords:** plant-based, sports nutrition, bodybuilding, muscle mass, strength, protein, leucine

## Abstract

Despite increasing awareness of plant-based diets for health and athletic performance, athletes are cautioned that careful dietary monitoring is necessary. Whether commonly consumed plant-based diets are nutritionally adequate for maximal muscular hypertrophy remains unknown. This modeling study assessed the nutrient composition of completely plant-based diets scaled to the caloric demands of maximal muscle mass and strength development in adult male bodybuilders. To model calorie requirements, anthropometric data from bodybuilders were input into the Tinsley resting metabolic rate prediction equation, and an appropriate physical activity factor and calorie surplus were applied. Dietary data from a large cohort following completely plant-based diets were then scaled to meet these needs. Modeled intakes for nutrients of interest were calculated as 1.8 g/kg/day of protein and 2.75 g/meal of leucine, which surpass mean requirements for maximal increases in muscle mass and strength and muscle protein synthesis, respectively. Daily levels for all micronutrients, except vitamin D, also exceeded requirements. Saturated fat levels were aligned with dietary guidelines, although sodium levels exceeded recommended limits. Consumption of larger portions of commonplace plant-based diets, scaled to meet the energy demands of maximal accrual of muscle mass and strength, satisfied protein and leucine requirements without the need for additional planning.

## 1. Introduction

A key motivation that drives the exercise habits of young men in the United States is muscle growth [1]. More than one quarter of this demographic pursues this goal, frequently engaging in muscle-enhancing behaviors including lifting weights, eating more food, and eating specific foods [2]. The position of the International Society of Sports Nutrition (ISSN) recommends animal-based protein sources for muscular hypertrophy [3], and this is reflected in the dietary habits of individuals undertaking resistance exercise training (RET) [4,5,6,7]. Poultry, dairy, eggs, and protein supplements constitute the main sources of protein in the diets of typical strength training gym users, and legumes may only be consumed rarely in this population [4]. Bodybuilders also consume large quantities of dietary protein [8], sourced mainly from meat and eggs [5,6,7]. Animal-based protein-rich foods and supplements may displace key nutrient-rich foods in these dietary patterns, where insufficient intake of fruits and vegetables has been reported in nearly half of gym users [9]. This contrasts with national public health recommendations that young men should decrease their intake of red and processed meat and include more fruits, vegetables, and whole grains in their diets [10] and international guidance to reduce meat intake and increase plant food [11,12]. There appears to be a disconnect between dietary recommendations for the amelioration of chronic diseases and increased muscular development, suggesting an either/or choice of long-term health or athletic pursuits.

At the same time, public awareness of plant-based diets for health and athletic performance has increased in recent years [13,14]. Plant-based diets encompass an array of dietary patterns that emphasize fruits, vegetables, whole grains, legumes, nuts, seeds, and plant products and may limit or exclude animal-derived products [15]. The term “completely plant-based diet” is applied in this study to describe diets that consist mainly of whole plant foods, reflected by fiber intakes that meet or exceed recommendations, and are devoid of animal products. Similar dietary patterns that have been previously studied use a variety of terminology, including vegan [16], “strict vegetarian” in the 7th Day Adventist Health Study [17], and whole food, plant-based [18]. The Academy of Nutrition and Dietetics has determined that plant-based diets, including vegetarian and vegan diets, are appropriate for athletes [19]. However, plant-based dietary patterns have historically been considered substandard for athletic performance and muscular development [20,21,22], with the primary reason being inadequate protein content [20,21,23,24,25,26]. Total protein intake is generally lower in people following plant-based diets compared to omnivorous diets [27], and plant proteins typically contain lower amounts of the amino acids (AAs) lysine, methionine, and leucine than animal-sourced proteins [28]. However, it is relevant to note that protein intakes generally exceed recommendations, regardless of diet type [27].

Leucine serves as both a building block for muscle protein synthesis (MPS) and a signaling molecule initiating MPS [29]. It has been determined that leucine is the most potent AA for stimulating MPS [30,31], which is the major driving force for nutrient-induced anabolism, processes involved in the creation of new bodily substances, cells, and tissues [32]. Caution, as well as careful planning and monitoring, has therefore been recommended to ensure that athletes following plant-based diets meet protein and amino acid needs [15,21,33,34,35,36]. Studies of aerobically or anaerobically trained adults who engage in recreational or competitive sports do not indicate inadequate intakes of total protein, lysine, methionine, leucine, key micronutrients, or lower diet quality, in individuals who follow plant-based diets compared to omnivorous diets [37,38,39,40,41,42]. However, these reports included subjects who consumed large quantities of protein supplements, obfuscating an evaluation of whether completely plant-based diets as commonly consumed (without emphasis on protein supplements) would satisfy the elevated protein required for maximal RET-induced gains in muscle mass and strength [43].

To the authors’ knowledge, the prevalence of plant-based dietary adherence in strength-sport athletes and those pursuing maximal muscle mass, such as bodybuilders, has not been reported. Bodybuilders are unique among strength-sport athletes in that the pursuit of muscle hypertrophy is the primary aim of RET, rather than a secondary consequence of strength and power development training [44]. The nutritional adequacy of completely plant-based diets followed by bodybuilders has been described [40], but plant-based diets prescribed for muscular hypertrophy frequently rely on protein supplements to maximize the anabolic response to RET [38,42]. It remains uncertain whether individuals who engage in RET and follow completely plant-based diets composed largely of whole foods that meet hypercaloric intake levels recommended to maximize muscle mass accrual (i.e., “bulking”) need to carefully plan and monitor their diets to meet protein, leucine, and micronutrient requirements.

For this modeling analysis, the nutrient needs of competitive bodybuilders were chosen because their professional success depends predominantly on their ability to maximize skeletal muscle mass [45,46]. Male bodybuilders were selected as the population of interest because female bodybuilders typically require less total protein intake due to their lower mean body mass [47,48]. There is a common belief that athletes and other individuals pursuing increased muscle mass while following plant-based diets require protein supplementation [49,50,51]. This modeling analysis aims to assess the validity of this perception. The objective of this modeling study is to determine whether commonplace, completely plant-based diets, scaled to meet the caloric needs of RET and muscular hypertrophy, provide sufficient protein and leucine to maximize muscle mass and strength while meeting micronutrient needs in male bodybuilders.

## 2. Materials and Methods

The estimated energy needs of competitive bodybuilders were calculated using data from a recent systematic review by Bauer et al. (2023) [48]. This systematic review included 16 studies that reported anthropometric data from 235 healthy male competitive bodybuilders with previous competition experience or currently in preparation for their first competition and who were not seeking weight loss [48]. The age range of these athletes was 22 to 35 years. Body mass and height ranged from 75 to 94 kg and 166 to 192 cm, respectively. Body fat levels were 5.8–10.7%. Weekly or monthly training durations, frequencies, and volumes were not described. However, other research has found that most competitive male bodybuilders completed 4–7 resistance training sessions per week, each lasting 60–90 min [52,53]. Off-season training sessions typically targeted 2–3 muscle groups, implemented 2–3 different exercises per muscle group, and included 3–4 sets per exercise. Sets were typically completed at intensities of 7–12 repetition maximum, and recovery between sets lasted 61–180 s. As shown in Table 1, median values were used for subsequent modeling calculations.

The Adventist Health Study-2 (AHS-2) cohort study was selected to provide dietary data for our model because it included the largest sample following a vegan diet (n = 5694) reported in the scientific literature [17]. In this study, the nutrient profiles of 71,751 subjects from AHS-2 were determined through a 204-item food frequency questionnaire (FFQ), which had been validated against 24 h recall data, and nutrition data were analyzed using a database containing over 20,000 foods [17]. Participants who reported consuming red meat, poultry, fish, eggs, milk, and dairy products not at all or less than once per month were classified as following strict vegetarian (vegan) diets. The mean fiber intake in this population was 46.7 g/day, which meets current guidelines [10] and thus satisfies our criteria for classification as a completely plant-based diet.

Energy requirements for maximal muscular hypertrophy can be calculated as total daily energy expenditure (TDEE) plus an energy surplus due to the whole-body anabolic response to overfeeding [54,55]. TDEE is comprised of the resting metabolic rate (RMR), which is the amount of energy required to maintain basic body functions, along with the appropriate physical activity level (PAL) factor [56]. RMR is calculated using body weight, age, and sex. Based on a recent meta-analysis evaluating the accuracy of RMR prediction equations in athletes [56], a newly developed RMR prediction equation was determined to be most accurate in male bodybuilders [57]. This formula, which is shown in Equations (1)–(3), was therefore used to calculate the predicted mean TDEE for the modeled sample.

Equations used to calculate energy requirements for male bodybuilders [54,55,57].

RMR (kcal/day) = 24.8 × Weight (kg) + 10(1)

ES (kcal/day) = RMR × PAL × 0.15 (2)

EER (kcal/day) = RMR × PAL + ES (kcal/day)(3)

EER: estimated energy requirements; ES: energy surplus; kcal: kilocalories; kg: kilograms; PAL: physical activity level; RMR: resting metabolic rate.

The determination of PAL was also necessary to calculate TDEE. During the off-season, or bulking phase, competitive male bodybuilders report engaging in four to seven days of RET per week, with session duration most commonly 60 to 90 min, in addition to one to four aerobic exercise sessions lasting 10 to 30 min [52]. This is reflective of a PAL of 1.75 (1.60–1.89), which is classified as “active” and describes athletes who exercise for approximately one hour per day [58]. Accordingly, a PAL of 1.75 was selected for calculations. It is worth noting that most bodybuilders reported completing twelve or more sets of exercise per muscle group per week [52]. This corresponds to a high training volume that has been established to stimulate maximal increases in muscle mass [59].

An energy surplus (ES) is recommended to achieve maximal hypertrophy because hypercaloric conditions potentiate muscle growth by supporting the increasing energy demands of progressively increasing RET volume [54,55]. A surplus of 10–20% of calories above maintenance needs has been recommended for more advanced trainees [55], the mean of which (15%) was used for calculations, as shown in Equations (1)–(3).

The determination of the protein intake level required to maximize RET-induced gains in muscle mass was based on a consensus statement from the International Olympic Committee (IOC) [60], which recommends protein intake for high-performance athletes pursuing maximal muscular hypertrophy and strength of 1.6 g/kg/day [47]. Daily protein intakes of 1.6 g/kg were therefore used for calculations. The mean protein intake in AHS-2 for subjects following a vegan diet was 14.5% of total calories [17]. EER were therefore multiplied by 0.145 to determine the mean calories from protein. This value was divided by four to calculate the mean total grams of protein.

To maximally trigger MPS, ISSN recommends leucine intakes as high as 3 g/meal [3], but other research suggests that this upper limit may be excessive [23]. Rather, dietary supplements containing 1.8 g of leucine appear to maximally stimulate MPS in young men and women [61,62]. When leucine content reaches or exceeds 2 g, as occurs with large protein doses, animal- and plant-based proteins exert similar anabolic effects [63,64,65]. We therefore set the threshold for effective leucine intake at 2 g per meal. It has been recommended that off-season bodybuilders consume 3–6 meals per day [66], and a simple solution for meeting protein and amino acid needs has been made to consume four protein-rich meals per day [67], one every three to four hours, to optimize MPS [68]. Consuming 2 g of leucine at each of four daily meals, for a total of ≥8 g per day of leucine, was therefore used as the target threshold for this modeling study. Leucine has been shown to constitute 8.8 ± 0.7% of animal-based proteins and 7.1 ± 0.8% of plant-based proteins [69], and dietary leucine content was calculated as 7.1% of total protein intake.

Target intake levels of saturated fat, ω3 polyunsaturated fatty acids (PUFA), linoleic acid, fiber, vitamin A, vitamin B6, folate, vitamin B12, vitamin C, vitamin D, vitamin E, calcium, iron, magnesium, phosphorus, potassium, sodium, and zinc were based on the Dietary Reference Intakes (DRIs) [70]. DRIs are nutrient reference values for vitamins and minerals intended to optimize health and prevent disease [71]. When available, Recommended Dietary Allowances (RDAs) were used. When unavailable, Adequate Intakes (AIs) were used. The sodium target intake level was based on the Chronic Disease Risk Reduction (CDRR) level [10]. Values pertaining to adult males aged 19–30 were selected for comparison with modeled intake levels in scaled plant-based diets.

## 3. Results

### 3.1. Energy Requirements

The estimated energy requirements were calculated, and the results are shown in Table 2.

### 3.2. Protein Requirements and Levels

Total daily protein requirements were calculated to be 135 g/day, as shown in Table 3. Total protein levels were calculated to be 151 g/day. Relative protein intake levels were then calculated to be 1.8 g/kg/day.

**Figure 1:**
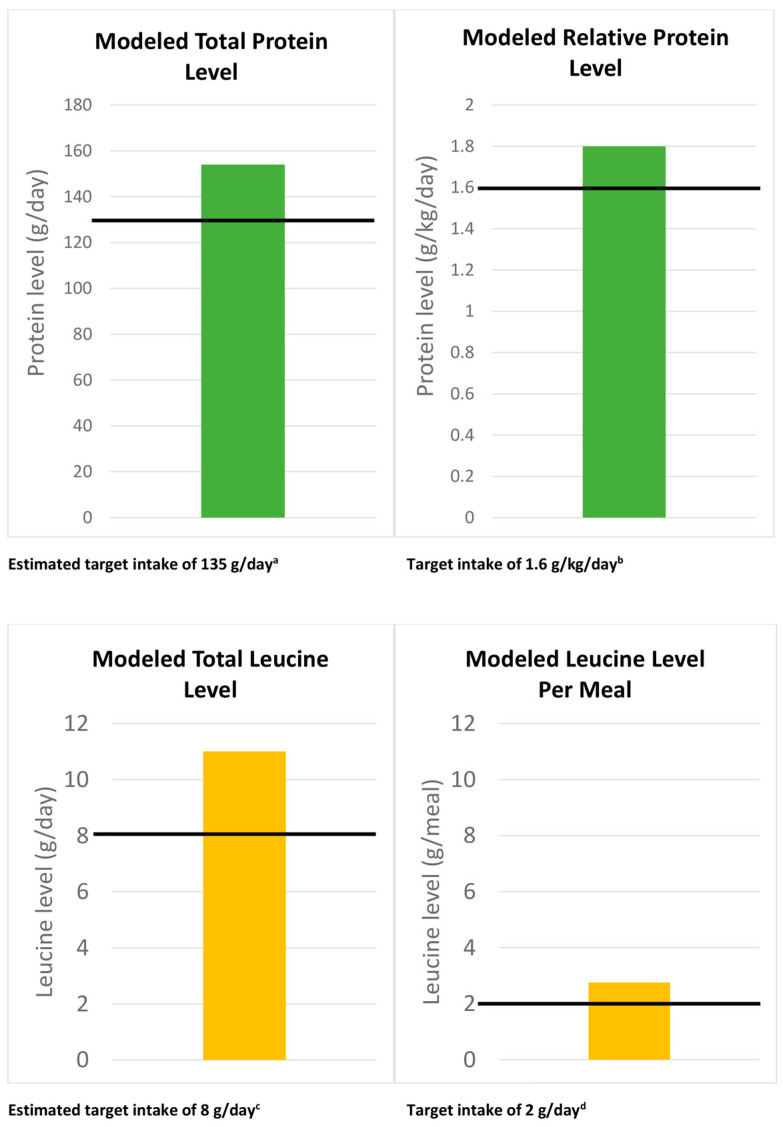
Protein and leucine levels scaled to meet the energy requirements of adult male bodybuilders following completely plant-based diets during the bulking phase, in relation to thresholds proposed to maximize gains in muscle mass and strength in response to resistance exercise training. g: grams; kg: kilograms. Footnotes: ^a^ Total protein requirements to maximize RET-induced increases in muscle mass and strength were determined as the product of 1.6 g/kg/day and mean athlete body mass in the population used for modeling [47,60]. ^b^ 1.6 g/kg/day was used as the target relative protein intake, based on a consensus statement from the International Olympic Committee [60], which recommends daily protein intakes for high-performance athletes pursuing maximal muscular hypertrophy and strength of 1.6 g/kg/day [47]. ^c^ Modeled total leucine target was set at 8 g/day because a solution to meet protein and amino acid requirements has been made to consume four protein-rich meals per day [67], one every three to four hours, to optimize MPS [68]. ^d^ Modeled leucine target was set at 2 g/meal because this quantity maximally stimulates MPS in young adults [61,62] and levels that reach or exceed this target exert similar anabolic effects regardless of whether they are sourced from animal- or plant-based proteins [63,64,65].

Total protein intake and protein intake levels relative to body mass exceeded target thresholds, as shown in Figure 1.

### 3.3. Leucine Levels

Leucine levels were calculated on a daily and per-meal basis, as shown in Equations (4) and (5). Both total daily leucine levels and leucine levels per meal exceeded target thresholds, as displayed in Figure 1.

Calculated leucine levels for male bodybuilders adhering to completely plant-based diets during the bulking phase.

Total leucine levels (11 g/day) = Total protein levels (151 g/day) × 7.1%(4)

Leucine per meal (2.75 g) = Total protein levels (11 g/day)/4 meals(5)

g: grams.

### 3.4. Micronutrient and Other Key Nutrient Levels

Average micronutrient intakes per 2000 kcal/day reported in the AHS-2 dataset [17] were scaled proportionately to 4239 kcal/day diets (Table 4). These values were directly compared to established DRIs for saturated fat, ω3 PUFA, linoleic acid, fiber, vitamin A, vitamin B6, folate, vitamin B12, vitamin C, vitamin D, vitamin E, calcium, iron, magnesium, phosphorus, potassium, sodium, and zinc [10]. All projected mean micronutrient levels met recommendations with the exceptions of vitamin D and sodium, as shown in Table 4. Projected mean vitamin D levels were slightly lower than the RDA (536 vs. 600 IU/day), and projected mean sodium levels exceeded the established CDRR level (7484 vs. 2300 mg/day). The projected mean saturated fat levels (5% of total calories) were below the established limits of 10% of total calories [10].

## 4. Discussion

This study assessed the adequacy of completely plant-based diets, tailored to the caloric demands of maximal skeletal muscle hypertrophy and strength development, in providing requisite amounts of protein, leucine, and essential micronutrients to adult male bodybuilders engaged in RET. Our findings substantiate that scaled plant-based diets satisfy protein requirements for maximizing RET-induced gains in muscle mass and strength. These diets also meet leucine thresholds and micronutrient adequacy, with the exception of vitamin D, for maximal stimulation of MPS and health requirements, respectively.

The daily energy requirements we calculated were larger than estimates in the existing literature. We calculated the calorie requirements for male bodybuilders to be 4239 kcal/day in the bulking phase based on the actual median age and body mass reported for these samples. This estimate exceeds findings from a systematic review of dietary intake studies in competitive bodybuilders following omnivorous diets that reported an average energy intake of 3821 kcal/day [8]. However, the dietary assessment methods used in these studies, which consisted mainly of 3- to 7-day food diaries, are known to underestimate energy intake [73]. Moreover, a recent cross-sectional study by Amatori and colleagues (2023) reported a mean calorie intake of 2632 kcal/day among bodybuilders following completely plant-based diets [40]. This study estimated food intake using a digital food diary, which may also underestimate energy intake [73]. This discrepancy may also be partially explained by differences in sex and body mass, which are variables within RMR equations that directly affect estimations [58]. Our modeling was performed using an entirely male population with a median body mass of 84.5 kg. Subjects in the studies by Spendlove (2015) and Amatori (2023) included 84% and 61% male subjects, and mean body masses were 83.7 kg and 72.3 kg in these samples, respectively [8,40]. The former reported mean body mass for men and women separately, and the latter reported mean body mass for men and women combined, which may have contributed to the divergent body masses and energy intakes reported in these studies.

The protein recommendations used in this study align with recommendations for maximal muscular development in young, healthy adults [43]. They align with existing guidelines for athletes following plant-based diets [74] and corroborate evidence that athletes undertaking RET while following completely plant-based diets meet protein requirements for maximal gains in muscle mass and strength [40,75,76]. Our study calculated protein intakes of 1.8 g/kg/day, which exceeds the mean intake levels of 1.6 g/kg/day required to maximize RET-induced gains in muscle mass and strength in healthy adults [47]. Protein intake levels were lower than those reported in a cross-sectional study by Amatori et al. (2023), which found that competitive bodybuilders adhering to completely plant-based diets consumed an average of 2.2 g/kg/day of protein derived from whole foods and supplements during the bulking phase [40]. Case reports have also indicated that well-trained bodybuilding and powerlifting subjects following plant-based diets achieved protein intakes ranging from 2.0–3.5 g/kg/day through a combination of whole foods and supplements [75,76]. Protein supplementation may explain the larger intakes reported in these studies as compared to the data analyzed by Rizzo et al. (2013) from the AHS-2 cohort [17], which included limited capture of some protein supplements or meal replacement shakes [77]. The top reported sources of dietary protein in this cohort among those following a vegan diet were whole plant foods such as legumes and grains [77]. However, the majority of research pertaining to protein intake for muscle protein remodeling has been conducted using protein supplements rather than whole foods, which may produce substantially different responses compared to whole food sources of protein [78]. Emerging evidence indicates that consumption of whole food protein sources effectively augments muscular development and recovery from exercise, but research is needed to further clarify the differential effects of protein from whole food and supplemental sources [79]. The modeled protein levels presented in this modeling study are generally achievable without protein supplements, however, and can be expected to meet the energy and protein needs of male bodybuilders.

Leucine intake was calculated due to its pivotal role in MPS [80]. Results indicated that scaled plant-based diets satisfied leucine requirements postulated to maximally stimulate MPS (2 g/meal) [63,64,65] by providing 11 g/day of leucine, or 2.75 g/meal, at each of four daily meals. These results align with data from the Oxford arm of the European Prospective Investigation into Cancer and Nutrition (EPIC–Oxford), in which young men who followed completely plant-based diets reported consuming a similar proportion of calories from leucine, scaling to 11 g/day if energy consumption reached the level determined for calculations in our study [81]. Similarly, Hevia-Larrain et al. (2021) found that young men who consumed 1.6 g/kg/day of protein while following completely plant-based diets and engaging in RET ingested an average of 9 g/day of leucine, with 2–3 g/meal consumed at breakfast, lunch, dinner, and evening snack [38]. When protein intake reaches this level, sufficient leucine has been consumed to maximize MPS, with additional intake producing no additional increases in RET-induced muscle mass and strength [82,83]. Furthermore, although plant-based proteins have been characterized as low in lysine and/or methionine relative to animal proteins, it has been suggested that large intake levels of plant proteins, as modeled in this study, would overcome these differences and produce a comparable adaptive response to RET [84].

This study also assessed micronutrient adequacy. Results indicated that scaled plant-based diets met recommendations for all vitamins, minerals, and essential fatty acids, with the exception of vitamin D. Vitamin D intake levels reached 536 IU/day, although 600 IU/day are recommended [10]. This finding aligns with lower intake levels observed in non-athletic and bodybuilding populations following plant-based diets [27,40], as well as bodybuilding populations following more standard diets [85]. Vitamin B12 is another established micronutrient of concern for individuals following plant-based diets [19], and projected intakes exceeded recommendations (49.4 vs. 2.4 mcg/day) [10]. These findings align with results from a recent cross-sectional study in which bodybuilders eating completely plant-based diets were reported to consume 50 mcg/day of vitamin B12 [40]. Conversely, Neufingerl et al. (2022) conducted a systematic review of nutrient intakes in non-athletic populations that reported mean vitamin B12 intakes in individuals following plant-based diets that were significantly lower (1.5 mcg/day) [27]. This may be attributable to relatively lower intakes of vitamin B12-fortified foods and beverages due to moderate energy intakes in more sedentary populations, as well as a lower prevalence of dietary supplementation in non-athletic populations [86]. Nevertheless, it can be challenging for bodybuilders to meet recommended intakes of vitamins B12 and D through fortified foods, and athletes are encouraged to ensure reliable intake of these micronutrients through the regular consumption of supplements [74,87]. Sodium intake levels (7484 mg/day) exceeded the upper limit set by the Dietary Guidelines for Americans (2300 mg/day) [10]. However, AHS-2 estimated dietary sodium using an FFQ [17], which has been shown to significantly overestimate these values [88]. Furthermore, fitness athletes sweat significantly during training, excreting approximately 1 g/hr of sodium during moderate-intensity sessions [89], which could affect sodium requirements in this population. Lastly, saturated fat was approximately 5% of total calories, which resides significantly below national recommendations to limit intake to less than 10% of total calories [10] and also meets the American Heart Association recommendations to limit saturated fat to no more than 5–6% of total calories for individuals with elevated low-density lipoprotein (LDL)-cholesterol levels [90]. This represents an improvement compared to traditional bodybuilding diets, which often contain larger quantities of saturated fat [8]. This is consequential because bodybuilders have displayed cardiovascular disease (CVD) risk factors [91,92] that may be largely attributable to diet [93]. However, the extents to which diet, chronic physical stress, and other factors contribute to the increased mortality rate associated with bodybuilding are not well understood [94]. Therefore, CVD risk in populations following completely plant-based diets compared to more typical, omnivorous diets followed by bodybuilding populations remains unknown and an area for future investigation.

The present study has several strengths. First, the sample size from which dietary intakes for a population following a completely plant-based diet was large [17]. Second, we used anthropometric data to derive energy requirements for a typical male bodybuilder [48] instead of relying on dietary assessment data for this population, which may be biased due to underreporting [95]. Third, the prediction equation used to estimate RMR has been validated in bodybuilders [57].

The study also has limitations. First, dietary intakes calculated in the AHS-2 cohort of individuals following completely plant-based diets were based on FFQ data, not more objective measures. FFQ are known to be subject to underreporting as compared to direct measures of actual intake [73]. However, because diets were scaled, it is possible this discrepancy did not affect the results. Second, we scaled dietary intake using modeling rather than direct laboratory analytical procedures, which could lead to discrepancies in projected versus actual intakes. For example, dietary fiber intakes were determined to be 99 g/day, and it has been speculated that consumption of fiber-rich foods could make it difficult for athletes requiring higher energy intakes to ingest sufficient calories due to the influence of fiber on satiety [33,35,96]. However, the selection of more calorically dense foods such as whole grains, nuts, and foods prepared with added fats as compared to fruits and vegetables alone may enable higher energy intakes. Additionally, the gastrointestinal tracts of athletes appear to be highly adaptable and permit larger calorie intakes by accelerating gastric emptying, diminishing perceptions of fullness, reducing bloating, improving tolerance to larger food volumes, and increasing absorption speed [97]. Third, modeling was performed for protein quantity but not protein quality, although this feature is more likely to influence anabolic outcomes at lower levels of protein intake than those determined in this study [63]. Popular means of calculating protein quality, such as the Digestible Indispensable Amino Acid Score (DIAAS), also display significant shortcomings that have been discussed in the context of plant-based diets [98] and limit their utility. Dietary leucine content was used to address this issue because a large body of evidence supports the leucine trigger hypothesis in explaining divergent postprandial rates of MPS in response to different protein sources [80]. The possibility exists, however, that the leucine threshold may only apply to the ingestion of isolated protein supplements rather than whole food protein sources due to differing digestion and absorption rates [80,99]. Following this, regardless of the total quantity consumed, leucine absorption and utilization may not be maximized if leucine intakes are not distributed evenly throughout the day [67,68]. However, the nutrient intakes of subjects from AHS-2 were not reported on a per-meal basis, so direct scaling of their meals may not optimally distribute their leucine contents. Additionally, a lack of dietary creatine has been proposed to limit the anabolic potential of plant-based diets [100]. We were unable to model dietary creatine intakes because levels are not reported in large cohort studies of populations following plant-based diets [27]. Although supplemental creatine has been shown to augment RET-induced increases in muscle strength [101,102] and hypertrophy [103], we have previously published that there is a lack of evidence indicating that dietary creatine also exerts these effects [100]. Finally, this study modeled levels of protein, leucine, and other key nutrients in the bodybuilding off-season, when calorie intake is high, relative to the dieting phase [8]. During contest preparation, 75% of competitive male bodybuilders have reported taking protein supplements [104], and it has been suggested that plant protein supplementation may be helpful for bodybuilders following plant-based diets since sourcing large quantities of protein from whole food sources would increase energy intake at a time when an energy deficit is required [40]. The results of this modeling study should therefore not be extrapolated to contest preparation.

This study modeled dietary intakes in young male bodybuilders following completely plant-based diets. Future research should determine protein intakes in older populations following completely plant-based diets. Protein requirements in older individuals may be greater than those in younger populations [105], although targets set in this study exceed these requirements [106]. Modeling protein intakes in female athletes would also be valuable. However, no differences have been observed between sexes regarding protein requirements beyond those related to differences in body mass for the maximization of RET-induced changes in muscular hypertrophy and strength [47]. Total energy and protein intakes in female bodybuilders are reportedly lower than in their male counterparts, likely due to their relatively lower body mass [8]. Additional research could model intakes among players of other sports following completely plant-based diets, in which levels of lean body mass are predictive of exercise performance [107,108,109,110]. Future research should also be performed using dietary data from populations outside the United States.

The findings of this modeling study have significant implications for the public health and fitness domains. They support a critical alignment between recommendations for the prevention and treatment of chronic diseases and the enhancement of muscle mass and strength in an athletic population. Dietary modeling suggests that athletes undertaking RET in pursuit of maximal gains in muscle mass and strength may meet dietary requirements by eating larger portions of commonly consumed plant-based meals.

## 5. Conclusions

This modeling analysis found that completely plant-based diets scaled to meet the caloric demands of maximal skeletal muscle hypertrophy and strength development in adult male bodybuilders satisfy protein, leucine, and essential micronutrient requirements necessary for maximizing RET-induced gains in muscle mass and strength. Our calculations indicate that these diets surpass the protein and leucine intakes required for optimal muscle development as recommended in existing literature, as well as most other micronutrient intake levels, with the exception of vitamin D. These findings indicate potential alignment between dietary guidelines for chronic disease prevention and sports nutrition requirements for maximal muscle hypertrophy and strength, bridging a gap that has previously separated these domains. This modeling research provides theoretical evidence that athletes adhering to completely plant-based diets can achieve their muscle-building goals while adhering to recommendations for overall health.

## Figures and Tables

**Table 1:** Physical parameters of male bodybuilders.

Parameter	Range	Median Value
Age (y)	22–35	28.5
Body mass (kg)	75–94	84.5

y: years; kg: kilograms.

**Table 2:** Calculated energy requirement calculations for male bodybuilders.

RMR: 2106 kcal/day
ES: 553 kcal/day
EER: 4239 kcal/day

ES: energy surplus; kcal: kilocalories; kg: kilograms; RMR: resting metabolic rate.

**Table 3:** Calculated protein requirements and levels for male bodybuilders adhering to completely plant-based diets during the bulking phase.

Total protein requirements: 135 g/day
Total protein levels: 151 g/day
Relative protein levels: 1.8 g/kg/day

g: grams; kg: kilograms.

**Table 4:** Scaled micronutrient and other key nutrient levels and requirements for male bodybuilders following completely plant-based diets.

	AHS-2 Strict Vegetarians	Male Bodybuilders ‡	Nutrient Target	Recommendation	Target Met?
Calories (kcal)	2000	4239	-	-	N/A
Saturated Fat (% kcal)	5	5	<10	DGA	✓
ω3 PUFA (g)	2	4.2	1.6	AI	✓
Linoleic Acid (g)	19.5	41	17	AI	✓
Fiber (g)	47	99	59 †	AI	✓
Vitamin A (mcg RAE *)	1108	2348	900	RDA	✓
Vitamin B6 (mg)	14.4	30.5	1.3	RDA	✓
Folate (mcg)	888	1882	400	RDA	✓
Vitamin B12 (mcg)	23.3	49.4	2.4	RDA	✓
Vitamin C (mg)	531	1125	90	RDA	✓
Vitamin D (IU)	252	536	600	RDA	✗
Vitamin E (mg)	101	214	15	RDA	✓
Calcium (mg)	1156	2450	1000	RDA	✓
Iron (mg)	32	67	8	RDA	✓
Magnesium (mg)	652	1382	400	RDA	✓
Phosphorus (mg)	1371	2906	700	RDA	✓
Potassium (mg)	4234	8973	3400	AI	✓
Sodium (mg)	3531	7484	2300	CDRR	✗
Zinc (mg)	16	35	11	RDA	✓

AI: Adequate Intake; CDRR: Chronic Disease Risk Reduction Level; DGA: Dietary Guidelines for Americans; DRI: Daily Recommended Intake; N/A: not applicable; ω3 PUFA: omega-3 polyunsaturated fatty acids; RDA: Recommended Dietary Allowance; RAE: Retinol Activity Equivalents. Data for AHS-2 strict vegetarians [17]. * Recommendations for Vitamin A provided in Retinol Activity Equivalents (RAE) to account for different bioactivities of provitamin A carotenoids [72]. † Based on recommendations to consume 14 g/1000 kcal [70]. ‡ Calorie intake represents the estimated energy requirements needed to maximize muscle hypertrophy for adult male bodybuilders. Mean micronutrient intakes per 2000 kcal/day reported in the AHS-2 dataset [17] were scaled proportionately to 4239 kcal/day.

## Data Availability

The original contributions presented in the study are included in the article.
